# 3D Printed Microfluidic Device for Magnetic Trapping and SERS Quantitative Evaluation of Environmental and Biomedical Analytes

**DOI:** 10.1021/acsami.1c09771

**Published:** 2021-07-14

**Authors:** Lucio Litti, Stefano Trivini, Davide Ferraro, Javier Reguera

**Affiliations:** † Department of Chemical Sciences, University of Padova, via Marzolo 1, 35131 Padova, Italy; ‡ Department of Physics and Astronomy, University of Padova, via Marzolo 8, 35131 Padova, Italy; § BCMaterials, Basque Center for Materials, Applications and Nanostructures, UPV/EHU Science Park, 48940 Leioa, Spain

**Keywords:** microfluidic, SERS, Janus nanoparticles, 3D printing, flumioxazin, erlotinib, partial least-squares regression

## Abstract

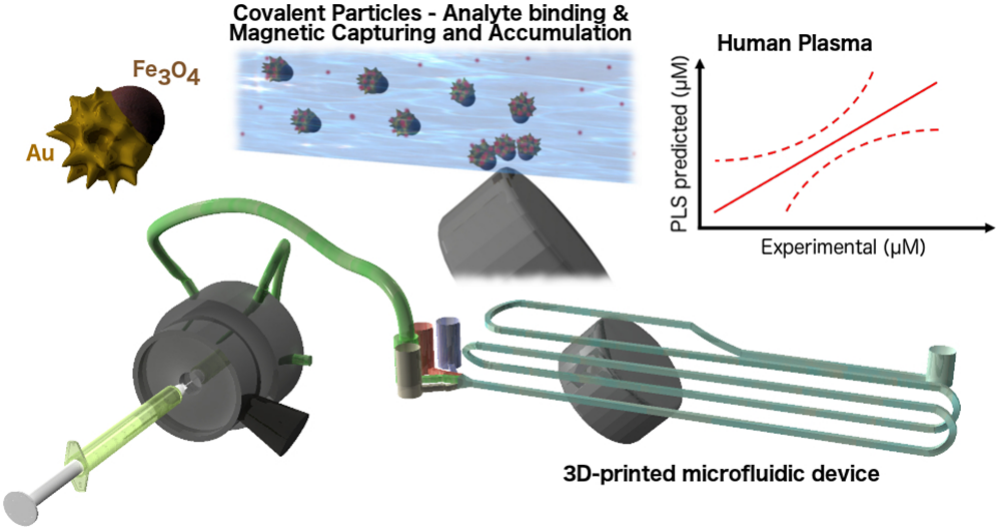

Surface-enhanced Raman scattering (SERS) is an ideal technique for environmental and biomedical sensor devices due to not only the highly informative vibrational features but also to its ultrasensitive nature and possibilities toward quantitative assays. Moreover, in these areas, SERS is especially useful as water hinders most of the spectroscopic techniques such as those based on IR absorption. Despite its promising possibilities, most SERS substrates and technological frameworks for SERS detection are still restricted to research laboratories, mainly due to a lack of robust technologies and standardized protocols. We present herein the implementation of Janus magnetic/plasmonic Fe_3_O_4_/Au nanostars (JMNSs) as SERS colloidal substrates for the quantitative determination of several analytes. This multifunctional substrate enables the application of an external magnetic field for JMNSs retention at a specific position within a microfluidic channel, leading to additional amplification of the SERS signals. A microfluidic device was devised and 3D printed as a demonstration of cheap and fast production, with the potential for large-scale implementation. As low as 100 μL of sample was sufficient to obtain results in 30 min, and the chip could be reused for several cycles. To show the potential and versatility of the sensing system, JMNSs were exploited with the microfluidic device for the detection of several relevant analytes showing increasing analytical difficulty, including the comparative detection of *p*-mercaptobenzoic acid and crystal violet and the quantitative detection of the herbicide flumioxazin and the anticancer drug erlotinib in plasma, where calibration curves within diagnostic concentration intervals were obtained.

## Introduction

Biosensing technologies often require quick, reusable, and cheap systems that allow a more general use where sampling is taking place. Among them, spectroscopic techniques are highly attractive, as they can be used in a relatively affordable manner, and they present a largely noninvasive character while providing insight into material composition, often with quantitative precision. Raman scattering and related techniques are exceptional spectroscopic techniques for the analysis of environmental or biomedical matrices, where water is overall the dominant component due to the low water Raman cross section.^1−5^ Notwithstanding, water is not the only species with a small Raman cross section, and thus, proper strategies are required to amplify the Raman signal from most analytes. Surface-enhanced Raman scattering (SERS), taking place mainly on the surface of nanostructured metals, overcomes the sensitivity drawback^2,5^ while offering a highly multiplexed analysis,^6^ intrinsically permitted from the recorded vibrational fingerprints, as shown for instance in complex confocal mapping.^7^ The process to upgrade SERS toward a reliable analytical technique is still challenging due to reproducibility issues,^8^ even if recent studies show promising results in developing standardized analytical SERS protocols,^9^ and their incorporation into easy-to-use and affordable devices.^10^

Microfluidics, on the other hand, has been successfully employed as a technological platform in many sensing techniques. It has also been applied in several SERS-based analytical devices, in which clear advantages can be recognized when the SERS substrate is not preincorporated in the chip, as it makes possible better and quicker mixing between SERS substrates (*i.e.*, particles) and analytes, allowing the integration of several processes typically performed using nanoparticles, e.g., chemical reactions, preconcentrations, purification processes, as well as improved reusability of the system.^11−21^ Additionally, SERS and microfluidics have been implemented for labeling and counting of cells and bacteria,^22,23^ in analogy to cytofluorimetry but replacing fluorescent labeling with SERS tags. On the opposite side, reaching a sufficient sampling speed, for instance, to measure thousands of cells in few minutes, requires extremely fast instrumentation^22^ or suitable strategies to slow down the flow while not affecting its dynamics.^24^ To overcome this problem, sandwich-like protocols have been designed to incorporate magnetic responsive particles with the help of iron oxide micrometric beads that can be accumulated under a magnetic field.^25^ Sophisticated magnetic/plasmonic nanostructures have been developed and used to target and isolate cancer cells, both *in vitro* and in microfluidic setups.^26−29^ Most of these applications, however, are still restricted to the detection of cells or bacteria, whereas a few examples can be found related to gas sensing,^30^ inorganic ions,^31^ and relatively small molecules such as food contaminants.^32^ General pros and cons of integrating SERS and microfluidics have been recently reviewed,^33^ concluding that SERS-based microfluidics is certainly not only attracting much attention but also encountering difficulties in transition from the laboratory level to the marketplace. As alternative SERS substrates, Au nanostars feature intrinsic hot spots located at the tips, thereby being advantageous with respect to spherical nanoparticles.^34,35^ Moreover, their combination with iron oxide, forming a small and compact hybrid nanomaterial, ensures efficient magnetic manipulation enabled by its superparamagnetic properties.^36−38^ The small dimensions as compared to the usually employed microbeads also allow a much faster diffusion and mixing with other reagents and analytes.^25,28,39^ This ensemble of properties makes nanohybrid magnetic and plasmonic SERS substrates an excellent candidate to be readily integrated into a microfluidic device, where an external magnetic field can be applied to remotely control nanoparticle accumulation and clearance. The challenge to translate quantitative SERS analysis toward practical applications may therefore take advantage of synergistic implementation of sophisticated magnetic/plasmonic nanomaterial into a compact microfluidic device. Proper surface functionalization should indeed guarantee the highest possible selectivity toward a specific target to overcome the limitation in complex matrixes, for example, blood or plasma. In this work, it is proven that a cheap and accessible 3D printed microfluidic chip can be used as a versatile SERS-based sensing device when used in combination with Janus magnetic/plasmonic nanostars Fe_3_O_4_/Au (JMNSs) that flow through the system, are magnetically accumulated, and allow magnetically enhanced SERS sensing. Their use is proven for several analytes in different matrixes and with increasing complexity and analytical difficulty. Several small-molecule analytes were selected for this study, including the standard pH-sensitive molecule *p*-mercaptobenzoic acid (MBA), a dye with antifungal and antibacterial activity (crystal violet, CV),^40^ a herbicide (flumioxazin, Flum),^41,42^ and a clinically relevant anticancer drug (erlotinib, Erl).^43^ Sensing of MBA and CV was intimal compared directly in solution and in the device. The results showed the amplification potential of the designed device and a completely different behavior between the two molecules attributed to their different adhesion chemical groups. Once the applicability was demonstrated with model molecules, similar comparatives (in solution (*in batch*) and in the microfluidic device (*in microfluidics*)) were applied to Erl and Flum, two more challenging molecules. To the best of the authors’ knowledge, not any Raman or SERS sensing device has been reported that achieves erlotinib quantification in human plasma or flumioxazin quantification in water. For their measurements, azide functionalization was performed on the nanoparticles. Sensing was then addressed based on click-chemistry-based capture and a competitive assay.^44^ Quantitative monovariate and multivariate calibration curves for all of such analytes were obtained from the SERS-based microfluidic platform using a limited sample volume, within the sub-micromolar concentration range and with a response time below 30 min. Limits of detection useful for diagnostics were obtained for the herbicide flumioxazin and the anticancer drug erlotinib, measured by human plasma samples. The results showed good measurement capacity of the system for pollutants and drugs in relevant biological/environmental ranges. Moreover, with respect to established techniques, like chromatography, the present technique showed similar amounts of sample and measuring time but without the requirement of organic solvents, with a minimal user manipulation, and being the overall 3D printed device simple, compact, and cheap (a few euros), which makes it attractive to translate into a versatile and accessible market device.

## Experimental Section

### Materials and Instruments

Sulfo-SANPAH was purchased from Thermo Scientific. Cysteamine (CAS 60-23-1), CV (CAS 548-62-9), MBA (CAS 1074-36-8), sodium dodecyl sulfate (SDS, CAS 151-21-3), flumioxazin (Flum, CAS 103 361-09-7), erlotinib (Erl, CAS 183 321-74-6), and solvents were purchased from Merk and used without further purification. Female human plasma (K2 EDTA), catalog # HMPLEDTA2-F, lot # BRH1413119, from Seralab, was used. Transmission electron microscopy (TEM) images were acquired with a JEOL JEM-1400PLUS microscope operating at 120 kV. For UV–vis–NIR spectra, an Agilent Cary 5000 was used. The Raman spectra were recorded with a Renishaw inVia μRaman system, equipped with a 785 nm laser excitation diode, a Peltier cooled CCD detector, and a Leica 10× magnification objective. Renishaw WiRE4 software and MATLAB R2019a were used for data treatment. A polynomial fit, by a built-in WiRE4 function, was used for baseline subtraction. A Cetoni neMESYS syringe pump system was used for microfluidic control, and a six-channel injection valve (usually adopted in HPLC instruments) was used for sample injection.

### JMNS Synthesis

The Janus nanoparticles were synthesized as previously described (complete description and characterization can be found in refs (45) and (46). Shortly, heterodimers of Au/Fe_3_O_4_ were first synthesized by reduction with oleylamine and thermal decomposition of Fe (CO)_5_ on the surface of presynthesized AuNP, giving rise to NPs of 5.7 ± 1.2 and 20.5 ± 4.0 nm in diameter for Au and Fe_3_O_4_, respectively. These heterodimers were used as seeds for the subsequent growth of Au nanostars (which grew from the Au part of the initial heterodimers, keeping the Janus configuration). A gold growth solution was previously prepared dissolving 4 g of polyvinylpyrrolidone (PVP) in 80 mL of *N*,*N*-dimethylformamide, wherein 0.436 mL of 50 mM HAuCl_4_·3H_2_O was added as soon as the polymer was dissolved. The solution was left to prereduce for 5 min (change from Au(III) to Au(I); the prereduction time depends on the PVP batch and was previously measured by UV–vis spectroscopy). The heterodimer seeds (0.8 mL, 1 mg/mL) were quickly added to the gold growth solution and left until the reaction was complete (in this case, they were left for 1.5 h). The nanostar size can be easily tuned by changing the amount of seeds added to the reaction. The NPs were then purified in four cycles of centrifugation/dispersion in DMF (and in water for the case of MBA and CV—in the following, we refer to this solution as the “JMNS stock solution in water”, at 1.3 mM (Au atom content)). TEM and UV–vis–NIR characterizations, reported in Figure S1, show that the nanostructures are star-shaped and show a localized surface plasmon resonance (LSPR) band centered at 740 nm, in close resonance with the 785 nm laser excitation used for Raman measurements.

### Azide-Functionalized Janus Magnetic/Plasmonic Nanostar (JMNS-N_3_) Synthesis

Surface functionalization of JMNSs was carried out following a recently reported method.^44^ JMNSs (1 mL), 9.5 mM Au content, in DMF were mixed with 50 μL of 3.38 mM cysteamine in DMF and gently stirred overnight. Subsequently, the mixture was centrifuged two times for 5 min at 6500 RCF and each time recovered in the same volume of fresh DMF. Therefore, 3.5 μmol of sulfo-SANPAH was solubilized in the minimum amount of DMF, added, and left under gentle stirring overnight. This step allowed producing an amide linked with the SANPAH molecule. The mixture was centrifuged 3 times for 5 min at 6500 RCF and each time recovered with 0.6 mM SDS. The colloid was finally diluted to 7 mL using 0.6 mM SDS for further use. In the following, we refer to this solution as the “JMNS-N_3_ stock solution in water”, at 1.3 mM (Au atom content). Surface functionalization was verified according to recently published procedures.^44^ The test for the success of functionalization is reported in the Supporting Information (Figure S8), in which two identical aliquots of JMNS-N_3_ and propynyl fluorescent red (PFR) were mixed in the presence, or not, of Cu^2+^ and sodium ascorbate solutions for the catalytic activation of the click chemistry reaction between N_3_ and PFR. As a negative control, the same reaction was used without the activating compounds. Each new batch was tested with the described quality checks and was found effective for up to several months when stored at 4 °C. Raman spectra were acquired without further purifications, and bright signals from PFR could be obtained only in the mixture in which the catalyst was present, meaning that PFR binds to JMNS-N_3_ due to the click reaction.

### Microfluidic Device Modeling and Fabrication

The microfluidic device, the base, and the magnet holder, with the relative dimensions shown in Figure S2, were designed using Blender 2.81 modeling software. All devices were made in transparent polycarbonate (PC) using an Ultimaker 2 extended^+^ 3D printer. The channels have a 1 × 1 mm^2^ section and a total volume of about 310 μL from the inputs to the measuring window (see Figures S2a and S3a). The measuring window was left open (*i.e.*, not printed) both at the top and the bottom and glued using Picodent Twinsil extrahard bicomponent resin with a glass coverslip and aluminum foil, respectively. The microfluidic device was designed to have four inputs: one for the JMNSs or JMNS-N_3_, one for pure water or, alternatively, for the sample, and the last two for the click chemistry activators (*i.e.*, Cu^2+^ and sodium ascorbate solutions) or for pure water in the case of JMNSs. A typical six-channel valve, equipped with a 100 μL loading loop, was integrated along the pure water line for easy sample injection. The elution profiles, summarized in Table S1 and used without changes for all measurements in this study, were optimized for the startup, sample elution, and device cleaning. The 3D printed device can be used for an indefinite number of cycles (all of the ones presented in this study, for instance), while the aluminum foil at the bottom of the measuring window was dismounted and cleaned occurently, about every four cycles on average.

### JMNS Assay with CV and MBA in Batch and within the Microfluidic Device

Stock solutions of CV and MBA were prepared in water at a concentration of 4 μM. The JMNS stock solution in water (500 μL) was diluted with 1.5 mL of 165 μM SDS. The assay *in batch* was performed by mixing 120 μL of analyte solutions (CV or MBA) with 100 μL of diluted JMNSs. The mixture was left under gentle stirring for 15 min, and then, Raman spectra were acquired from the liquid sample without further purification (785 nm excitation, 10× objective, 60 mW). Three measurements were acquired and averaged; the error bars represent the standard deviation. The assay within the *microfluidic* device was also carried out using solutions of CV and MBA, injected through the six-channel valve equipped with a 100 μL charging loop. The elution programs are reported in Table S1 for all measurements. The amount of JMNSs was 100 μL for each test, and the extra two syringes were filled with pure water. When the elution program was finished, a SERS map (785 nm excitation, 10× objective, 30 mW, single scan at 3 s acquisition for each spectrum, 200 × 200 μm^2^ map, with a total of 45 spectra) was acquired around the spot made by the particles attracted by the magnet, and the spectra obtained directly over the spot were averaged; the error bars represent the standard deviation. Finally, the magnet was removed, and the device was cleaned by the “cleaning” elution program (see Table S1). All Raman spectra were baseline-subtracted, a Fourier transform noise filter was applied, and characteristic bands were integrated and plotted against the analyte concentration.

### JMNS-N_3_ Assay with PFR, Flum, and Erl in Batch and within the Microfluidic Device

Stock solutions of Flum and Erl were prepared in ethanol at a concentration of 20 μM for both. The JMNS-N_3_ stock solution in water (500 μL) was diluted with 1.5 mL of 165 μM SDS. PFR was synthesized as previously described, and a stock solution of 6.9 μM in ethanol was used.^44^ The assays in solution (*in batch*) were performed by mixing 120 μL of diluted solutions of Flum, or Erl, with 30 μL of 6.9 μM PFR, 100 μL of diluted JMNS-N_3_, 100 μL of 2 mM CuSO_4_, and 100 μL of 17 mM sodium ascorbate. The mixture was left under gentle stirring for 15 min, and then, Raman spectra were acquired from the liquid sample without further purification (785 nm excitation, 10× objective, 60 mW, single scan at 10 s acquisition for each spectrum). Three measurements were acquired and averaged; the error bars represent the standard deviation. The assays within the microfluidic device were carried out using dilute dilutions of Flum and Erl, 120 μL added to 30 μL of 6.9 μM PFR, injected through the six-channel valve equipped with a 100 μL charging loop. For human plasma samples, aliquots of Erl were added to 100 μL of plasma to reach concentrations within the micromolar range, which are the concentrations used for the plots in Figure 4. Then, 200 μL of EtOH was added, followed by 10 min centrifugation at 0 °C. The supernatant (120 μL) was mixed with 30 μL of PFR in EtOH, and then, the sample was injected into the device through the 100 μL loop. The elution programs were those reported in Table S1 for all measurements, namely, the amounts of JMNS-N_3_ were 100 μL for each test and the other two syringes were filled with 2 mM CuSO_4_ and 17 mM sodium ascorbate. At the end of the elution program, a Raman map (785 nm excitation, 10× objective, 30 mW, single scan at 3 s acquisition for each spectrum, 200 × 200 μm^2^ map, with a total of 45 spectra) was acquired around the spot of particles attracted by the magnet, and the spectra obtained directly over the spot were averaged; the error bars represent the standard error. Finally, the magnet was removed, and the device was cleaned by the “cleaning” elution program. All Raman spectra were baseline-subtracted, a Fourier transform-based noise filter was applied, and partial least-squares (PLS) regression was applied using the build-in *plsregress* MATLAB function under the tenfold cross-validation property. In total, 95% prediction bands were calculated by the build-in *polyval* MATLAB function, for the linear correlation between the experimental analyte concentrations and the PLS-predicted concentrations.

### BEM and DFT Simulations

Boundary element method simulations were performed using the MNPBEM toolbox developed by Hohenester,^47^ and nanostructure geometries were designed using Blender 2.81 software. Both the extinction cross section and the locally enhanced electric field (expressed as the fourth power and referred to as the SERS enhancement in the following) were calculated using an excitation field propagating and polarized along *x*, *y*, and *z*, and then averaged. DFT simulations of flumioxazin Raman spectra were performed using Gaussian software and the B3LYP/6-311+G(d,p) functional.

### Microfluidic Device Simulations

3D finite-element simulations (by Comsol Multiphysics) have been performed to analyze (i) the mixing efficiency in the microchannel and (ii) the magnetic field gradient generated by the permanent magnet. The first allows evaluating when the complete mixing of the liquids injected at the four entrances is achieved to choose the correct positioning of the permanent magnet or the imposed flow (see Figure S2). Then, the second set of simulations aims to optimize the orientation of the magnet with respect to the microchannel in which the JMNS flow (see Figure S3 for more details).

## Results and Discussion

JMNSs were synthesized as previously described (see Experimental Section).^36^ The synthesis resulted in 70 nm nanostructures (Figure S1d), as identified from TEM images (Figure S1c), where the smaller and lower contrast Fe_3_O_4_ lobes are visible upon close inspection (light gray features in Figure S1e). The Janus character of these nanoparticles has been reported earlier using EDX mapping and electron tomography, regardless of the nanostar size.^46^ The vis–NIR extinction spectrum of JMNSs presents a maximum at about 740 nm, which correlates with the LSPR tip mode of an ensemble of randomly oriented Au nanostars (Figure S1a). The same nanostar ensemble was used to calculate the SERS enhancements when excited at 785 nm, as shown in Figure S1b. One can see that the JMNSs tips act as *hotspots*, enhancing the local field by several orders of magnitudes without the need for aggregating them as is the case with spherical plasmonic nanoparticles.^34,35^ The advantages provided by the magnetic properties of JMNSs were reported in previous studies.^36,45,46,48^

### 3D Printed Microfluidic Device

**Figure 1:**
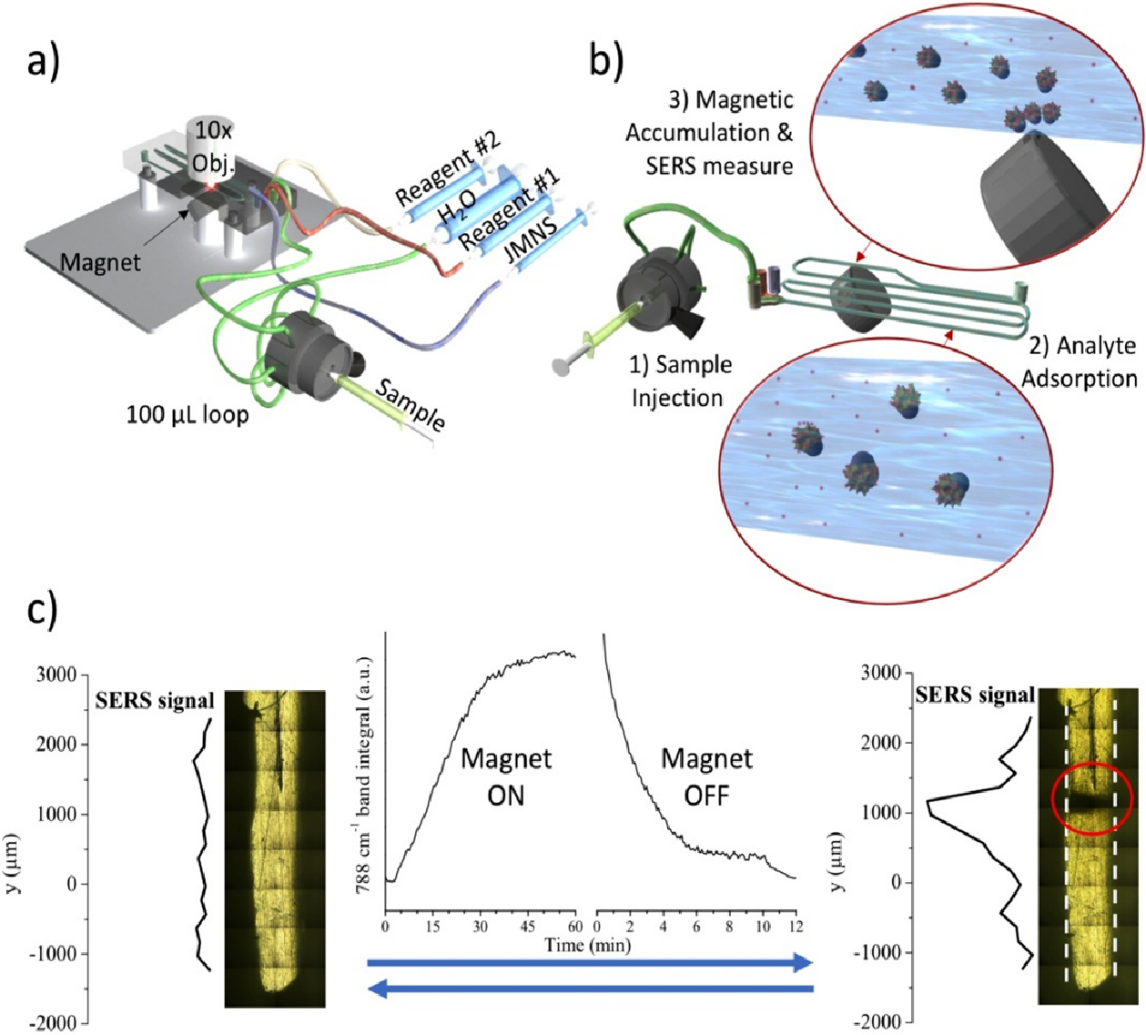
(a) Microfluidic device was devised to be highly compact. The plate was sized to take place under the μRaman microscope and to perfectly orient the magnet holder at the center of the channel. The six-channel injection valve was integrated into one of the four independent lines. (b) Scheme representing the assay within the microfluidic device. The sample is injected through a six-channel valve. Along the channels, the analytes (red dots) react and adsorb on the JMNSs that are, finally, magnetically retained due to their superparamagnetic Fe_3_O_4_ lobe. (c) Two pictures of the channel (about 1 mm wide) corresponding to the measurement window in the 3D printed microfluidic device. Thirty minutes were required to reach the maximum SERS signal, for the defined elution programs. The red circle on the right image marks the spot generated by the JMNSs accumulated by the magnet at the bottom. The SERS signal profiles represent the SERS intensity recorded along the channel length and evidence a maximum located at the JMNSs accumulation point, which can be completely cleaned in about 10 min. The SERS signals belong to PFR molecules bound to the azide-functionalized JMNP.

A 3D printed microfluidic device was herein designed integrating a section in which the particles would accumulate under the effect of a magnetic field gradient.^49^ Finite-element simulations provided evidence for the highest achievable magnetic field gradient, and thus, the highest magnetic force driving nanoparticle accumulation is reached by placing the magnet at 45° with respect to the flow direction (Figure S3). The device, printed in PC according to the geometry depicted in Figures 1a and S2, was devised to have four inputs for the nanoparticles, the sample, and two other entries (reagents or buffers, for instance). The geometry of the four inlets assures the mixing of solutions, estimated to complete between 5 and 10 cm (about one-fourth of the overall length, at which the magnet is placed), from the inlets (Figure S3a–c). To allocate the device in the μRaman microscope’s plate, a base was also 3D printed. The base has also the function to host the holder for the magnet, ensuring its alignment at the center of the channel in the measurement window (Figure S1c–f). A typical six-channel valve, equipped with a 100 μL loading loop, was integrated for sample injection. Once initialized, the system is completely closed and semiautomated for elution and data recording. The last ensures a consistent practical advantage as, once the device is set up, it stays closed and insulated. Not any risk of bubble insertion can occur during the changes between different samples because they are injected directly through the dedicated valve into one of the four inlets, as represented in Figure 1a. Additionally, being the microfluidic device entirely printed as a single part, eventual risks of delamination and unbonding issues are prevented.^50^

Once the sample is injected within the device, analyte molecules are free to adsorb on the surface of JMNSs. The mixture then reaches the measurement area, where the particles are attracted and retained by the magnet at the bottom of the channel, *i.e.*, at a single spot, prior to running SERS measurements (Figure 1b). After each analysis, the magnet can be removed, and the particles are washed out of the device. Figure 1c reports nanoparticle accumulation and cleaning kinetics, with Raman measurements obtained at the same channel position. Examples on the reusability of the device, alternating measuring and cleaning cycles, are reported in Figure S4. The elution profiles are resumed in Table S1 and designed to reach the maximum nanoparticle accumulation (namely, the associated asymptotic SERS signal in Figure 1c) within 30 min, as a fair balance between fast recording and sufficient time for the analytes to reach adsorption equilibrium with the JMNSs. On the other hand, a separate cleaning cycle was also optimized, much faster than the previous one, and to be applied once the magnet is removed. Both of them allow completing an entire circle of elution, magnetic attraction, Raman measurement, and device cleaning within less than 1 h.

### Direct Detection of CV and MBA

**Figure 2:**
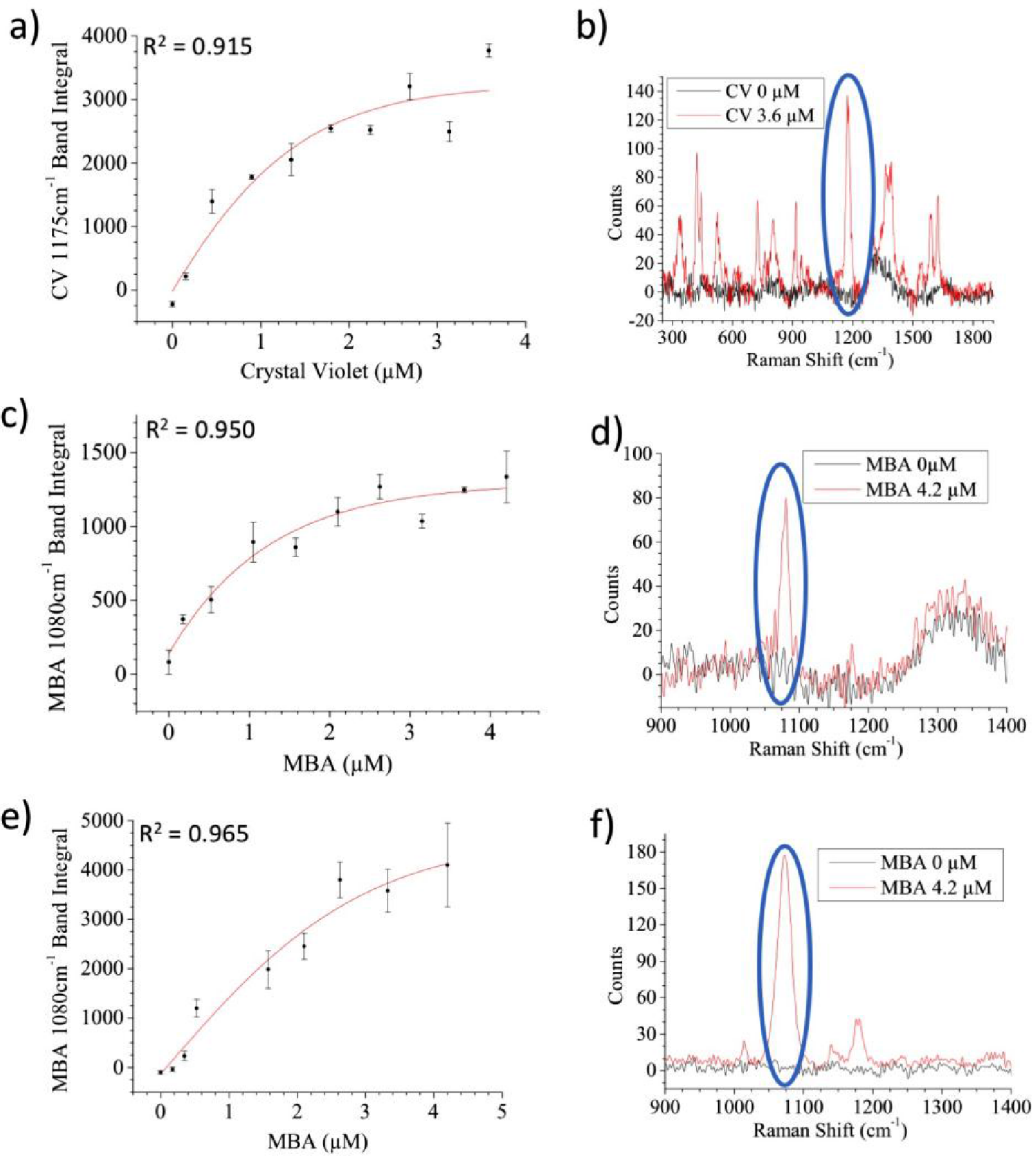
(a and c) Plots of the SERS intensity of characteristic peaks for CV (a) and MBA (c), at a fixed amount of JMNSs and increasing dye concentration, obtained in batch. (b and d) SERS spectra at maximum and minimum concentrations are reported in plots (a) and (c) for CV and MBA, respectively. (e) SERS intensity for increasing concentrations of MBA, after mixing with JMNSs in the microfluidic device followed by magnetic accumulation. (f) Two SERS spectra obtained from solutions of panel (e). Blue circles in (b), (d), and (f) correspond to the selected peaks for integration. Error bars represent standard errors.

The ability of JMNSs to adsorb analytes and generate quantitative curves through their SERS signals was tested and compared using *batch* and *microfluidic* experiments. A *batch experiment* is defined as the situation where all of the reagents and the analyte are directly mixed in a vial, and the mixture is left to react and then measured as it is. In the *microfluidic experiment*, one should consider the system described in the previous section, where all of the reagents are preloaded in a closed microfluidic system and the samples are injected through a six-channel valve. In the latter, the SERS measurement is obtained by the magnetically accumulated JMNSs, and then the device is cleaned before the next sampling. JMNSs were therefore first tested *in batch*, with increasing amounts of CV and MBA, whose molecular structures are reported in Figure S5. The SERS spectra were measured in solution, with an 785 nm laser excitation. From the characteristic spectral features of the two analytes, shown on the right panels in Figure 2, one specific band, at 1175 cm^–1^ for CV and 1080 cm^–1^ for MBA, was selected to monitor the band integral profile at increasing concentrations. CV is a dye commonly used for SERS experiments in the NIR region.^10,51^ High-intensity signals were therefore recorded, and a calibration curve was obtained in the micromolar and sub-micromolar range (see Figure 2a,b). MBA is far less bright in terms of Raman/SERS cross section,^35,51^ but it ensures strong adsorption onto the Au surface of JMNSs due to Au–thiol binding. Figure 2c,d shows the results obtained by mixing JMNSs and MBA *in batch*, confirming that a calibration curve can still be retrieved within the same concentration range of CV. A Boltzmann sigmoidal function was chosen as a base for empirically fitting the calibration curves (see eq 1, where *A*_1_, *A*_2_, *x*_0_, and d*x* are left as free parameters). The observed sigmoidal is consistent with a complete surface coverage of JMNSs (especially the *hot spots*) as the upper limit of the achievable signal^29^
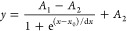
1

The curves for both CV and MBA were obtained again using the *microfluidic* device and magnetic preconcentration prior to Raman measurements. This protocol was first used to analyze the amount of CV in an aqueous solution. In this case, even if JMNSs were correctly accumulated on the magnet, the CV SERS signals could not be detected (Figure S6). On the contrary, MBA signals were clearly recovered (Figure 2e,f). This can be rationalized by the weaker interactions of CV with JMNSs, in comparison to the stronger Au–thiol interaction of MBA. In the case of CV, the initial adsorption on the JMNP in solution is followed, after accumulation by the magnet, by desorption due to the washing effect of the solution flowing in the channel. This result demonstrates the importance of stronger linking between the analyte and the SERS substrate^52^ for flow devices; the following implementation is a direct consequence of this evidence, namely, of providing the strongest possible interaction between the JMNSs and the analytes.

### Competition Assay for Flumioxazin and Erlotinib

**Figure 3:**
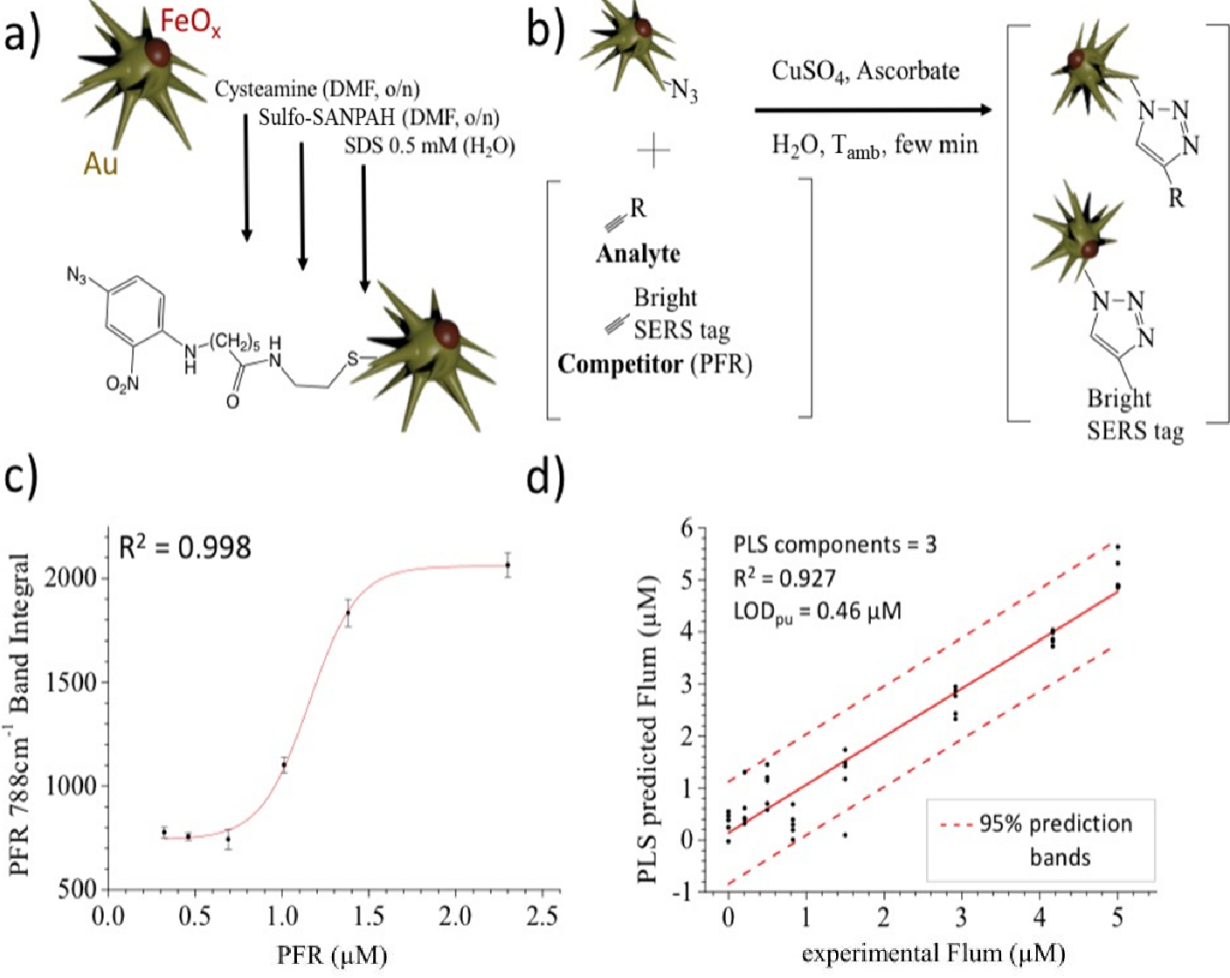
(a) Functionalization steps for the synthesis of JMNS-N_3_. (b) Scheme of the Huisgen click reaction on the surface of JMNS-N_3_. (c) PFR and JMNSs were mixed within the microfluidic device and the SERS spectra were recorded after magnetic accumulation. (d) Competition assay curve for flumioxazin (Flum) within the microfluidic device. PFR SERS signals were used to indirectly monitor flumioxazin concentration using the pseudounivariate PLS regression curve.

The second set of experiments with this sensing device was first implemented through the detection of flumioxazin, a herbicide frequently applied to the soil with severe effects on plants at the nanomolar concentration range.^42^ Both the European Union and the United States proposed a maximum level of residues, for several fruits and vegetables, of 0.02 ppm and identified the lowest relevant acute toxicity by oral administration at 2.2 mg/kg of body weight per day.^53,54^ We first performed DFT simulations of the Raman spectrum of flumioxazin, and the results (Figure S7a and Table S2) were in good agreement with the Raman spectrum measured from flumioxazin powder. In flumioxazin, any strong binding site for the surface of JMNSs, as thiol groups in the case of MBA, is not present (Figure S5) and low Raman cross section hinders its detection by direct SERS spectroscopy, in which any of the predicted Raman bands cannot be detected (Figure S7a). Situations like the present case, namely, when the analyte has neither strong affinity to the plasmonic surface nor high Raman cross section, are particularly challenging and one may suppose that SERS will never be successfully applied.^55^ To overcome this limitation, advanced surface functionalization can be engineered, in which a selected target may preferentially bind, therefore enhancing its adsorption in respect of other interferences. The performance of such kind of approach was recently reviewed, and detections down to the trace level were achieved for a wide range of environmental and biomedical analytes.^56^ We applied a slightly different strategy based on a competitive reactive SERS approach, in which the analyte molecule reacts with the surface of JMNSs in competition with another molecule, with a much stronger SERS cross section than the analyte and therefore easier to detect.^44^ The analyte concentration is consequently indirectly monitored through the competitor signals, overcoming the limitations introduced above. In this case, one chooses a specific common reaction for both molecules, which in the present case is a catalyzed azide–alkyne Huisgen click reaction, activated by the presence of Cu^+^ ions obtained *in situ* by the reduction of Cu^2+^ with ascorbic acid. The so-called competitive assay sacrifices the possibility of multicomponent analysis due to direct analyte’s vibrational fingerprint detection but compensates for the possibility to obtain calibration curves with high signals by species with intrinsic low Raman/SERS performances. The insurgence of false positives, due to the concomitant presence of species with acetylenes groups, is a calculated risk as acetylene is a relatively unusual functional group in natural products.^57^ To provide the proper receptor on the JMNSs for both the analyte and the competitor, the particles were functionalized with azide groups (−N_3_) (see Experimental Section and Figure 3a) considering that both the analyte and the competitor molecules show an alkyne group (C≡C). Therefore, the competitive assay proceeded through a click-chemistry reaction between the target analyte (flumioxazin^54^ in this case) and a second high-cross-section molecule, which competes for the same sites on the surface of JMNS-N_3_ (Figure 3b). The selected competitor molecule was PFR (cf. Figure S5), a dye modified with an acetylenic group that shows bright SERS spectra when excited at 633 or 785 nm.^44^ A SERS analysis of JMNP-N_3_ and PFR alone (namely, without Flum) was first carried out *in batch* to evaluate the JMNS-N_3_ and PFR reaction kinetics (Figure S8b). It was also tested that no signal from PFR can be detected by only mixing the JMNS-N_3_ and PFR in the absence of the catalysts (*i.e.*, copper salt and ascorbate solutions, Figure S8a,b), meaning that the selectivity given by the click reaction is transferred to SERS detection. Finally, it was successfully verified that increasing amounts of PFR provide consequent increases of its characteristic SERS signals (Figure S9a,b). The same procedure was implemented within the microfluidic device under magnetic attraction for the JMNS-N_3_/PFR conjugate, where Cu^2+^ and ascorbate solutions occupied the remaining two input flow lines. The elution program (Table S1) provided sufficient time for the click reaction to reach the maximum yield (Figure S8b) and allowed the magnetic sample concentration at the measuring window, resulting in a signal gain of a factor of about 3 (Figure S8c), with respect to the same reaction without the microfluidic device. The calibration curve for PFR within the microfluidic device is reported in Figure 3c (spectra are reported in Figure S9c) and shows a sigmoidal profile within the micromolar and sub-micromolar range. Experiments with flumioxazin were obtained with a constant amount of PFR. The PFR dose was chosen in the range near the saturation observed in the curve of Figure 3c to maximize the SERS signal and render the system highly responsive. The competitive assay, thus, consists of mixing a constant amount of JMNS-N_3_, PFR, CuSO_4_, and ascorbic acid and a variable amount of the analyte solution (flumioxazin solution in this case). The detection of increasing amounts of fluomioxazin, in a wide concentration range, was clearly observed with decreasing SERS signals of PFR in *batch* (Figure S7). Flumioxazin was further analyzed in *microfluidic experiments*, where JMNSs, PFR, CuSO_4_, and ascorbic acid are flown through the different inlets due to the programs reported in Table S1, and the sample is injected through the online six-channel valve to another inlet. The inverse sigmoidal shape of the response curve is converted in the so-called pseudo-univariate correlation through PLS regression.^58^ Generally speaking, PLS is one of the most popular multivariate models to achieve correlations by reprojecting the data into a set of new axis components ordered in decreasing sample representativity (variance explained). In PLS, the components are also built to maximize the correlation between the observations (spectra) and the queried property (concentration). The first few components are consequently used to build a prediction model for, as in the present case, the analyte concentration in an unknown sample. The most important requirement to properly apply PLS is to set an adequate number of components to be used for the prediction model. Few components may result in inaccurate predictions; too much components will overfit the data. The so-called leave-one-out cross-validation is a common approach to choose the proper number of components, checking the first components that minimize the estimated mean-squared prediction error (MSE). The build-in *pslregress* MATLAB function was used under the tenfold cross-validation property for the MSE estimation. The PLS pseudounivariate curve was obtained for the competitive assay of flumioxazin, in the microfluidic device, using the first three components (Figures 3d and S10a for the MSE) with high *R*^2^ and a pseudo-univariate limit of detection (LOD_pu_) of 0.46 μM, calculated as suggested by Allegrini et al.^58^ Importantly, the obtained sensitivity was found sufficient to monitor acute toxicological exposure, as defined by EU laws.

**Figure 4:**
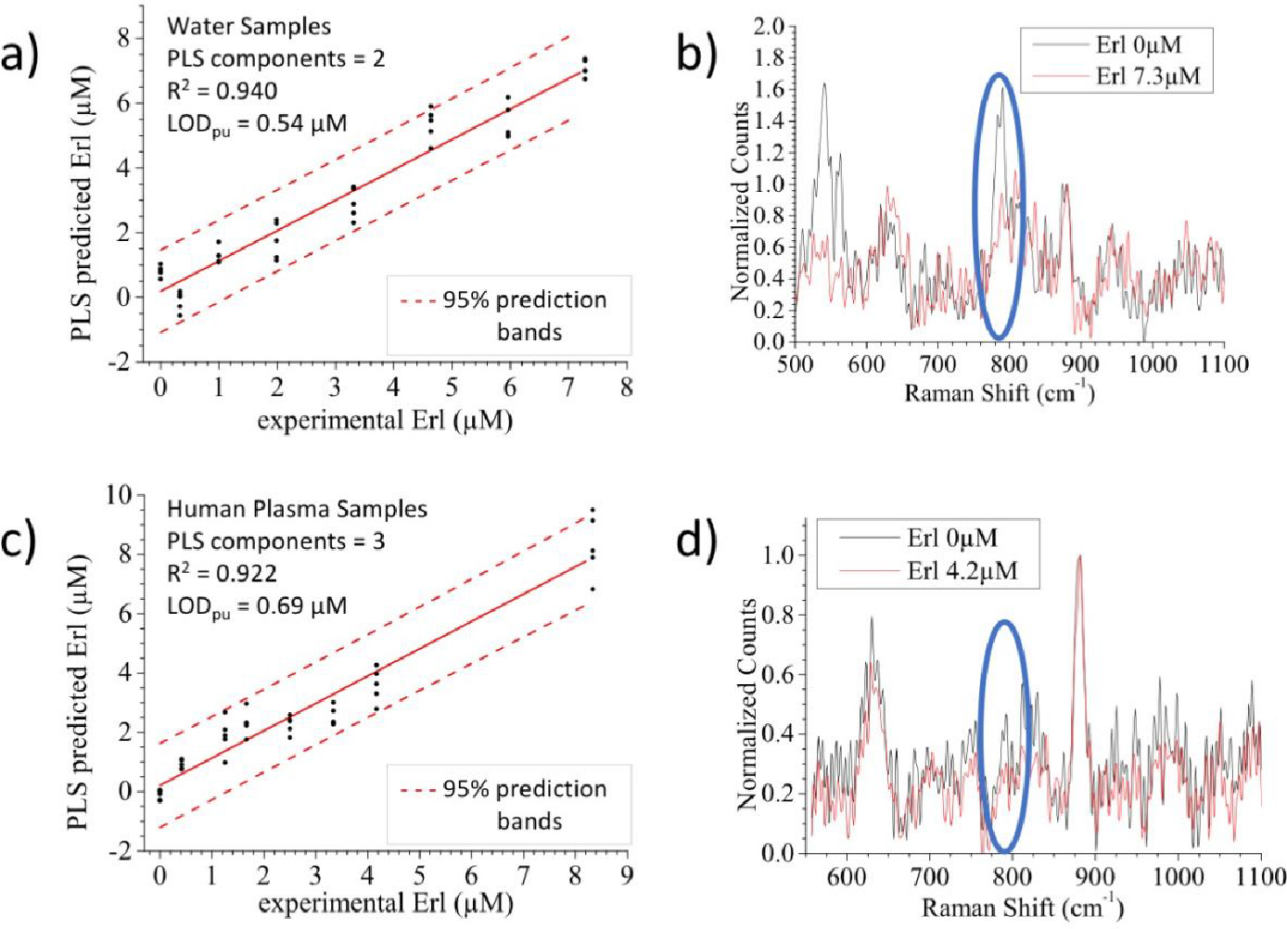
PLS competition assay pseudounivariate curves obtained for erlotinib within the microfluidic device. (a and b) Water samples spiked with erlotinib and spectra related to the two curve limits and (c and d) human plasma samples spiked with erlotinib and spectra related to the two curve limits. The blue circles in (b) and (d) indicate one of the most characterizing PFR bands that decreases with an increased erlotinib concentration due to the competitive assay.

As a second example of the application of the microfluidic device with the competitive reactive SERS approach, the detection of the anticancer drug erlotinib in human plasma was performed. Erlotinib has an alkyne group, and it has been previously inspiring the competitive reactive SERS approach but in a static solution setup.^44^ The pharmaceutical activity of this drug involves the inhibition of kinase enzyme at the epidermal growth factor receptor (EGFR) protein, and therefore, it is used to treat several types of cancer, including pancreatic cancer.^44,59^ The usual steady-state concentration of erlotinib in patients’ blood has been reported to be within the micromolar range.^44,59^ As just mentioned for flumioxazin, the presence of other molecules with acetylene groups may result in false positives. Nevertheless, even if it is a moderately common functional group in medicinal chemistry,^60^ it may not be considered as a critical issue, as the type and amount of drugs containing acetylene groups administered to a patient is known *a priori* and the amount of endogenous molecules containing acetylene groups is limited and at much lower concentration than the applied drug. Figure 4a,b shows the pseudounivariate PLS regression curve of the competitive assay of erlotinib in water using the microfluidic device. The decreasing trend of the PFR band signal (550 and 788 cm^–1^, Figure 4b), upon increasing the amount of erlotinib, can be clearly observed in a similar way to the previous examples with flumioxazin (Figure S7d). On the contrary, the bands at about 630 and 860 cm^–1^ appear to remain unaltered, as they can be ascribed to oxidized ascorbic acid^61^ and ethanol (Figure S7a). In this case, two PLS components were used for the prediction model (Figure S10b) and a LOD_pu_ of 0.54 μM was estimated. The competitive assay was then tested in a more complex and realistic medium, such as blood plasma. The spiked samples were prepared by adding known amounts of a concentrated stock solution of erlotinib directly to human plasma. A sample pretreatment, with the addition of ethanol to produce mild protein depletion, was adopted prior to injection in the microfluidic system. The data reported in Figures 4c,d and S11 show that a calibration curve can be obtained also for erlotinib in plasma, although with SERS signals of smaller intensities and a LOD_pu_ shift to 0.69 μM for erlotinib (again three PLS components used, Figure S10c). These two effects (lower signals and higher LOD_pu_) are due to the dilution caused by plasma pretreatment and to the presence of residual proteins in the plasma matrix, which, being not completely removed, can bind to the nanoparticle causing a protein-corona effect and a decrease in the yield of the click reaction.^44^ Despite a calibration curve is obtained within the micromolar range, so within the clinically relevant concentration range for erlotinib, there is still room for improving the overall signal intensities. The results obtained with the microfluidic device and the magnetic/plasmonic nanoparticles are relevant as they demonstrate how the magnetic accumulation of the nanoparticles allows recording more intense spectra than in the case of the solution conducted procedure, namely, the batch experiments without the present device.^44^ Moreover, to the best of the authors’ knowledge, not any successful SERS sensing device has been reported before that addresses erlotinib quantification in human plasma samples.

## Conclusions

In conclusion, Janus magnetic/plasmonic nanostructures were exploited in a 3D printed microfluidic device for quantitative evaluation of relevant substances for environmental and biomedical applications. The device was demonstrated to be cheap (less than 5 euros each device), of limited dimensions, with easy sample injection, and it was shown to give quantitative results within short times. Importantly, the possibility of using the device more times after the final cleaning step gives it further value. The small quantity of the sample needed for the measurements (100 μL) and the overall procedure lasting less than 40 min are other interesting characteristics of the device. The quantitative evaluation of flumioxazin, a widely used herbicide, was obtained, for the first time, with a competitive reactive SERS approach. Erlotinib, a clinical anticancer drug, was also detected in the spiked sample of plasma, which is an important result for clinical applications. PLS regressions were involved to obtain the best calibration of the PFR signal over the analyte concentration and to establish the LOD. The limited dynamic range achieved is a direct consequence of the amount of particles used as well as general optimizations that will follow for the analytical validation. Nevertheless, all of the analyses resulted in calibration curves on the useful concentration interval for practical applications. The present approach constitutes a step forward for the development of trustworthy analytical methods and technological solutions based on SERS.

## Supporting Information Available

Additional TEM and spectroscopic nanoparticle characterization; elution profiles; microfluidic device details, sizes, and pictures; microfluidic simulations; additional Raman spectra; and DFT Raman simulation of flumioxazin (PDF)
