# Supplementary material for: 3D Printed Microfluidic Device for Magnetic Trapping and SERS Quantitative Evaluation of Environmental and Biomedical Analytes
Source: ACS Appl Mater Interfaces. 2021 Jul 14;13(29):34752–61. doi: 10.1021/acsami.1c09771 (PMC8397251; doi:10.1021/acsami.1c09771)
Supplement: Supplementary file 1 — am1c09771_si_001.pdf [file am1c09771_si_001.pdf]

Supporting Information

3D printed microfluidic device for magnetic trapping and SERS quantitative evaluation of environmental and biomedical analytes

Lucio Litti,[a] Stefano Trivini,[a] Davide Ferraro,[b] Javier Reguera [c*]*

a) Department of Chemical Sciences, University of Padova, via Marzolo 1, 35131 Padova, Italy
b) Department of Physics and Astronomy, University of Padova, via Marzolo 8, 35131 Padova, Italy
c) BCMaterials, Basque Center for Materials, Applications and Nanostructures, UPV/EHU Science Park, 48940 Leioa, Spain

* lucio.litti@unipd.it; javier.reguera@bcmaterials.net

Stefano Trivini current address: CIC nanoGUNE, Tolosa Hiribidea 76, 20018, Donostia, Spain

TEM images about the Janus character of the Magnetic/Plasmonic Nanostars (Figure S1e)

The large size of the nanostars in comparison with the small 15 nm iron oxide lobes covers its visualization in the TEM 2D projection image. However, these small lobes have a much lower contrast (light gray) and are visible in some nanoparticles that are facing laterally. On the other hand, when the nanoparticles are facing vertically, the lower contrast of the Fe_3O_4 can be perceived as a cavity in the center of the nanoparticle. Additionally, on some occasions, the initial gold seed (~5-6 nm) can be also perceived in those cavities. The position of the Fe_3O_4 has been marked, when visible, with an arrow to help its identification in the image. This Janus morphology is in agreement with the previously reported characterization of the nanoparticles by TEM of series of nanoparticles at varying nanostar size and by electron tomography of individual nanoparticles.

References:

- *Reguera, J., et al. (2016). "Synthesis of Janus plasmonic–magnetic, star–sphere nanoparticles, and their application in SERS detection." Faraday discussions 191: 47-59.*
- *Reguera, J., et al. (2017). "Janus plasmonic–magnetic gold–iron oxide nanoparticles as contrast agents for multimodal imaging." Nanoscale 9(27): 9467-9480.*

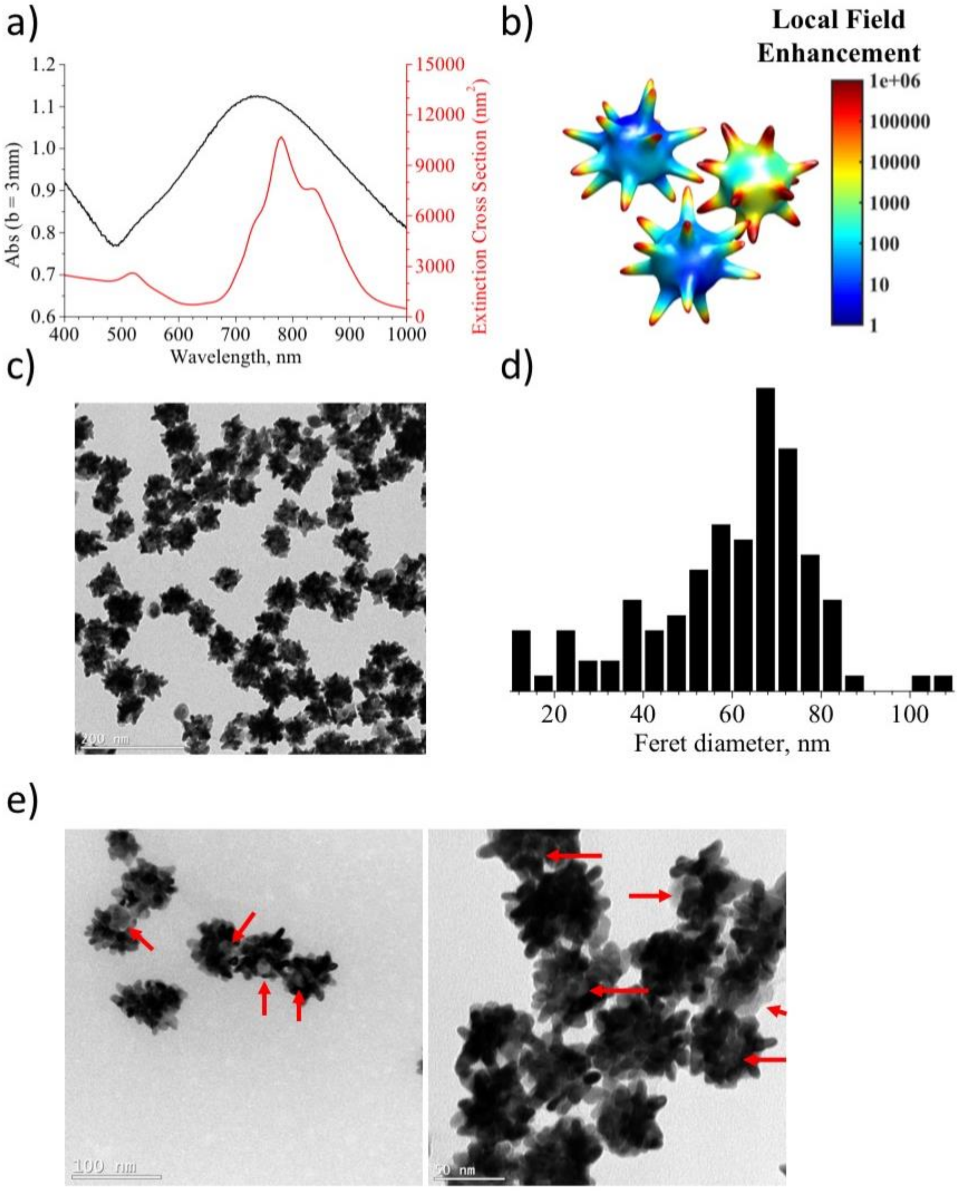

Figure S 1: a) vis-NIR extinction spectrum of JMNS and BEM simulated extinction cross-section of the ensemble composed of three JMNS. b) the same JMNS ensemble was simulated for the representation of the local field enhancement when excited at 785 nm. c-d) TEM image of JMNS and corresponding size distribution. e) TEM images of Janus nanoparticles of Au (nanostar):Fe_3O_4 (nanosphere), the last is marked with red arrows when visible.

Table S1: the three elution programs. In the "Time, min" column the total time of the process is reported adding subsequently the time required by each step.

<table>
<tr><th colspan="6">STARTUP</th></tr>
<tr><th colspan="2">Time, min</th><th colspan="4" rowspan="2">Flow, µL/min</th></tr>
<tr><th>Total time</th><th>Step time</th></tr>
<tr><td></td><td></td><td>JMNS</td><td>Reagent #2</td><td>H_2O (Sample)</td><td>Reagent #1</td></tr>
<tr><td>0</td><td></td><td rowspan="2">10</td><td rowspan="2">10</td><td rowspan="2">10</td><td rowspan="2">10</td></tr>
<tr><td>1</td><td>1</td></tr>
<tr><td></td><td></td><td rowspan="2">0</td><td rowspan="2">0</td><td rowspan="2">300</td><td rowspan="2">0</td></tr>
<tr><td>11</td><td>10</td></tr>
</table>

<table>
<tr><th colspan="6">INJECTION – Sample Elution</th></tr>
<tr><th colspan="2">Time, min</th><th colspan="4" rowspan="2">Flow, µL/min</th></tr>
<tr><th>Total time</th><th>Step time</th></tr>
<tr><td></td><td></td><td>JMNS</td><td>Reagent #2</td><td>H_2O (Sample)</td><td>Reagent #1</td></tr>
<tr><td>0</td><td></td><td rowspan="2">0</td><td rowspan="2">0</td><td rowspan="2">25</td><td rowspan="2">0</td></tr>
<tr><td>17''</td><td>17''</td></tr>
<tr><td></td><td></td><td rowspan="2">25</td><td rowspan="2">25</td><td rowspan="2">25</td><td rowspan="2">25</td></tr>
<tr><td>3'17''</td><td>3</td></tr>
<tr><td></td><td></td><td rowspan="2">5</td><td rowspan="2">5</td><td rowspan="2">5</td><td rowspan="2">5</td></tr>
<tr><td>8'17''</td><td>5</td></tr>
<tr><td></td><td></td><td rowspan="2">0</td><td rowspan="2">0</td><td rowspan="2">20</td><td rowspan="2">0</td></tr>
<tr><td>23'17''</td><td>15</td></tr>
</table>

<table>
<tr><th colspan="6">CLEANING</th></tr>
<tr><th colspan="2">Time, min</th><th colspan="4" rowspan="2">Flow, µL/min</th></tr>
<tr><th>Total time</th><th>Step time</th></tr>
<tr><td></td><td></td><td>JMNS</td><td>Reagent #2</td><td>H_2O (Sample)</td><td>Reagent #1</td></tr>
<tr><td>0</td><td></td><td rowspan="2">0</td><td rowspan="2">0</td><td rowspan="2">300</td><td rowspan="2">0</td></tr>
<tr><td>10</td><td>10</td></tr>
</table>

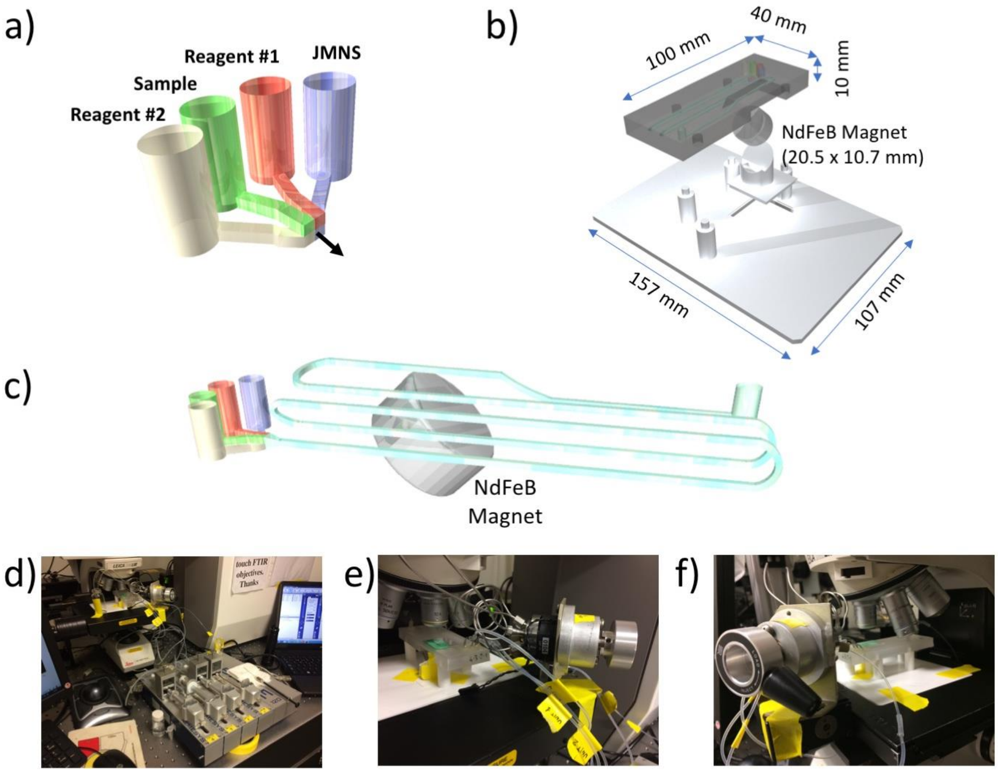

Figure S 2: a) the 3D printing approach allows to design a convergent flow for the four maximum inputs; b) the design is compact in dimension and made of several modules to increase its flexibility; c) Insight on the channels and magnet configuration; d-e-f) Photos of the experimental setup showing the pump, the injection valve, and the printed device under the Raman microscope.

Microfluidic Simulations

Before flowing at the proximity of the external magnet, the liquid flows along 340 mm in the channel. This long channel design assures the efficient mixing of the four aqueous solutions. As a matter of a fact, as shown in the simulation reported in **Figure S3**, the complete mixing is achieved before 100 mm from the liquid inlets, assuming the worst mixing conditions (perfectly smooth surfaces and without turbulence at the inlets cross). The simulation is set in order to mix four different solutions presenting increasing concentration (from 0, 250, 750 and 1000 mol/m^3) of a hypothetical solute. Therefore, when the concentration of 500 mol/m^3 is reached, the complete mixing of the four liquids is assured. In detail, the coefficient diffusion of water is used for all four liquids, while the lowest flows reported in **Table S1** are set at the channel inlets. **Figure S3c** reports that the mixing is achieved between 50mm and 100 mm from the channel junction (0 mm in the x-axis). **Figure S3d** reports the results of the simulation of the magnetic field generated by the magnet. **Figure S3e** shows the measured (black square) and simulated (red dots) value of the magnetic field B as a function of the distance from the magnet along the magnetization direction. The perfect overlap between the data assure the good quality of the simulation in order to replicate the experimental condition. In the 3D printed structure, the magnet has been installed tilted at an angle of 45 degrees with respect to the microfluidic channel (see **Figure S3d**) in order to ensure a high magnetic field gradient and thus, a high magnetic force. This is confirmed by the simulation results reported in Figure S3e, in which the magnetic field measured along the 45° direction (blue) is sharper than those along the magnetization axis (red and black). Therefore, magnetic field grandient (∇B) considered along the 45° direction results much higher than the values found on the magnetization axis, up to 3 mm from the magnet (see **Figure S3f**). In detail, at 0.5mm (see dash line in **Figure S3f**), corresponding to the distance between the magnet and the microchannel the ∇B is one order of magnitude higher in the first case than in the second (i.e. 0.315 T/mm and 0.034 T/mm, respectively). The same ratio is expected for the magnetic force (Serra, M., et al. Lab on a Chip 2017, 17(23), 3979-3999).

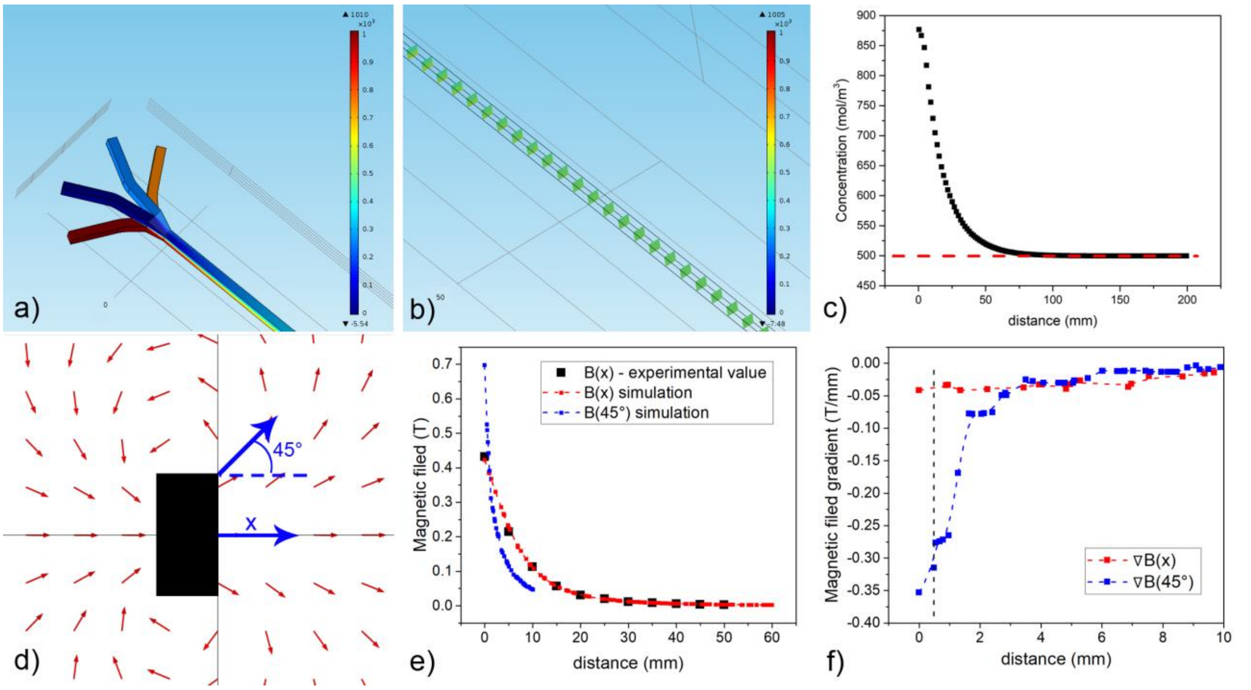

Figure S 3: a) the four inlets of the microfluidic device were assumed to contain four different aqueous solutions at different concentrations of the same analyte. b) once the concentration in the squared section of the channel reaches a uniform value it can be considered that the perfect mixing occurs. The figure legend reports a color scale of the hyphotetical concentrations. c) Plot of the concentration along a line placed at the bottom of the microfluidic channel; the calculated data show a complete mixing between 50 and 100 mm from the inlets at the present flow conditions. d-f) Simulation of the magnetic forces to which the JMNS are subjected once they reach the part of the channel over the permanent magnet. The higher magnetic field gradient along the direction placed at 45° from the magnetization axis justifies its choice for the placement of magnet with respect to the section of the channel.

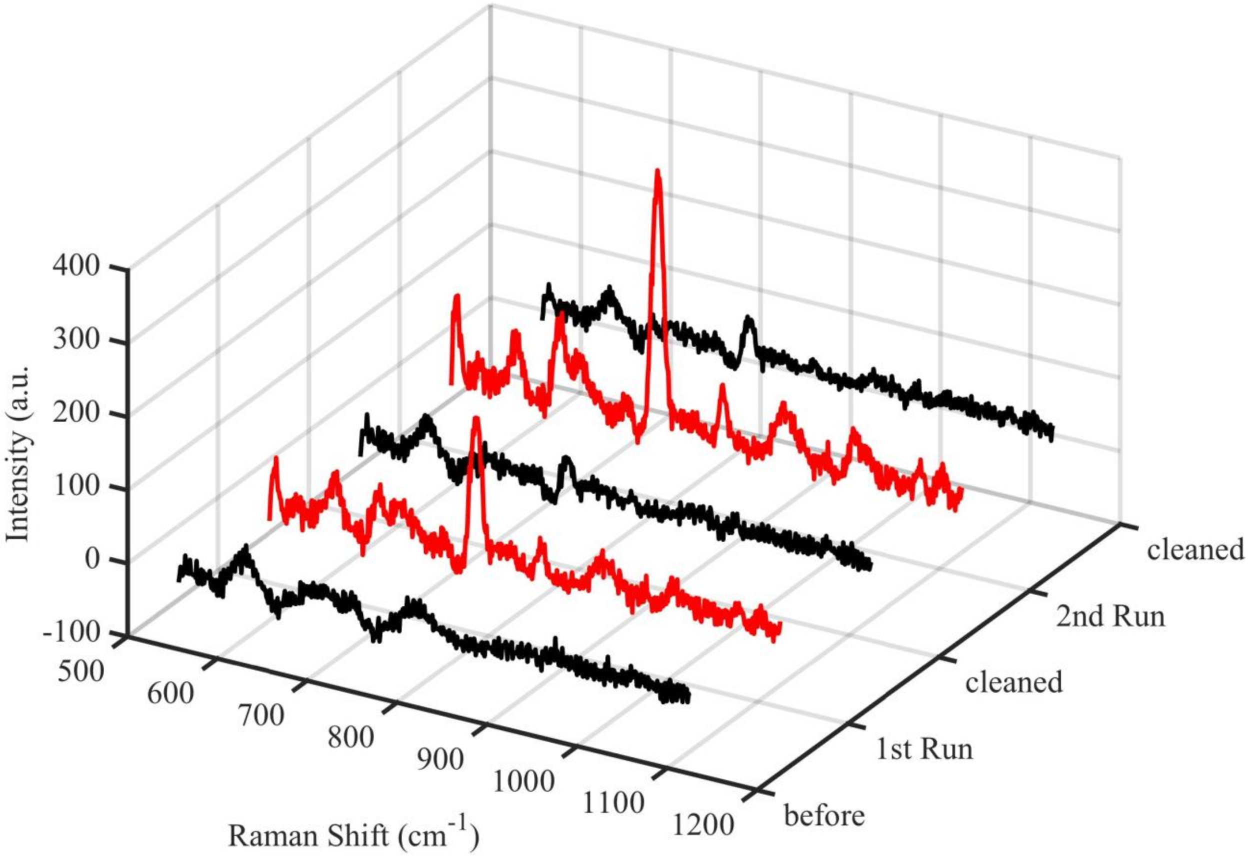

Figure S 4: Series of spectra obtained on the same position in the measuring window of the microfluidic device, representing two consecutive elution cycles of magnetically retained JMNS. It can be seen that the characteristic spectrum of the functionalized JMNS is almost entirely cleaned after the magnet is removed and the cleaning cycle (refer to Table S1) adopted.

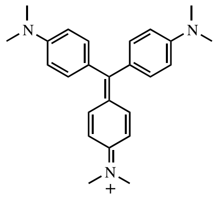

Crystal Violet

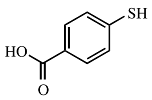

MBA (p-mercaptobenzoic acid)

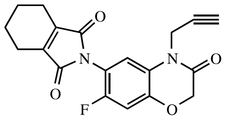

Flumioxazin

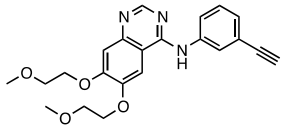

Erlotinib

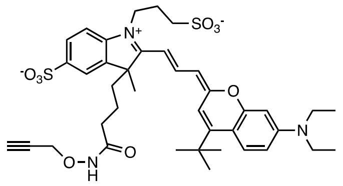

PFR (Propynyl Fluorescent Red)

Figure S 5: Molecular formula of the species analyzed in the study.

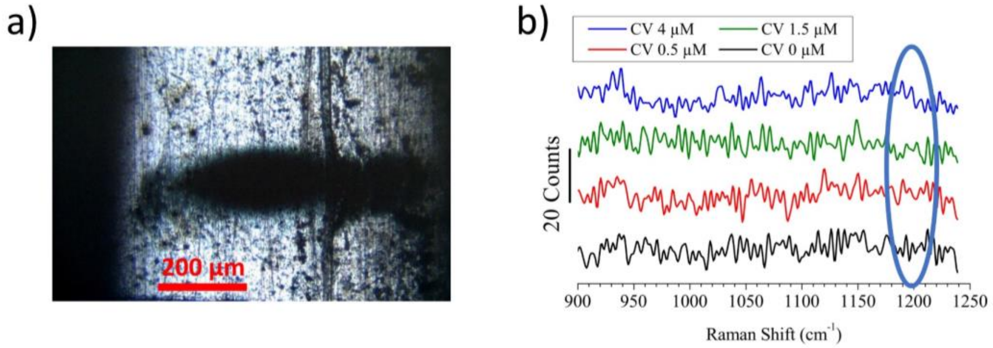

Figure S 6: JMNS and CV interaction in the microfluidic device. a) the particles are correctly accumulated in correspondence to the magnet location but b) no clear band from the Crystal Violet can be identified.

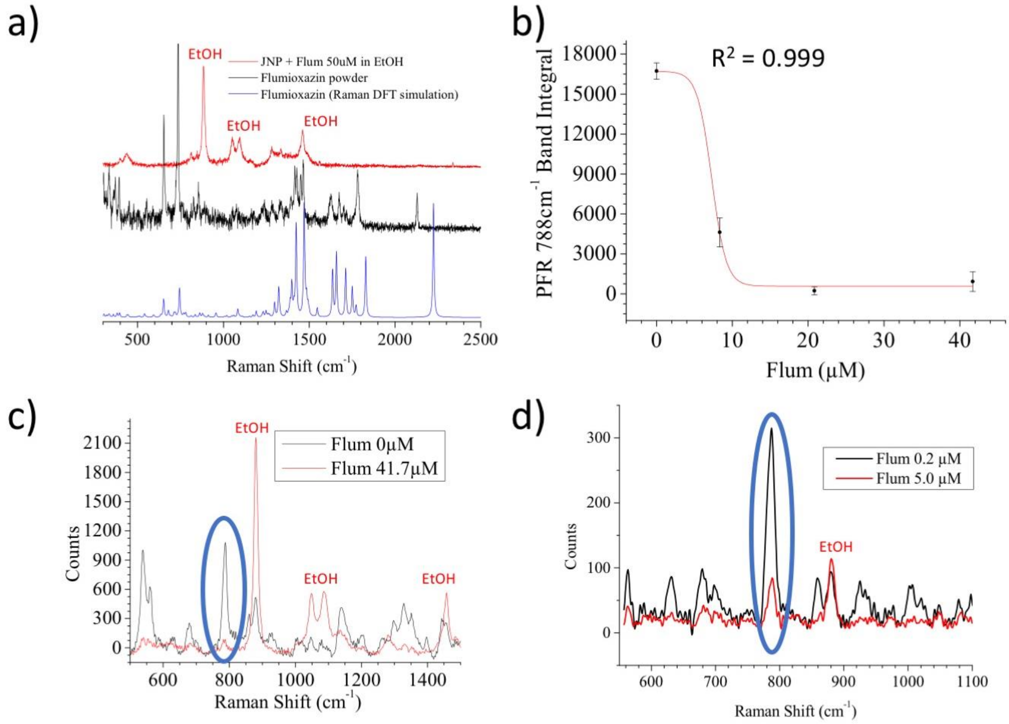

Figure S 7: a) A solution of Flumioxazin was mixed with JMNS, but no clear band can be detected, in comparison to the spectrum obtained by Flumioxazin powder and the simulated Raman spectrum; b) The competitive essay of Flumioxazin against PFR conducted in solution shows the expected inverse sigmoidal trend, error bars represent the standard errors; c) two spectra from the tests of panel b; d) two spectra from the competitive essays of Flumioxazin against PFR conducted in the microfluidic device.

Table S2: DFT Raman simulation of Flumioxazin, obtained with Gaussian using the B3LYP/6-311+G(d,p) functional. In the following, the main peaks of Figure S6a are reported with the relative comparison with the experimental spectrum and related attribution. Functional group indexes are referred to the structure reported below.

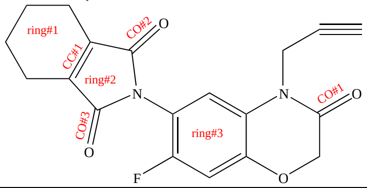

Frequency, cm^{-1} DFT	Frequency, cm^{-1} Experimental, powder at 785nm	Attribution
652.45	653	ring#1 & #2 breathing
744	735	ring#3 breathing
1398.17	1395	amines asym. stretch.
1423.93	1415 - 1428	amines sym. stretch.
1469.61	1450	CH ring#1 bending
1471.07	1465	CH ring#1 bending
1636.25 - 1658.74	1626	ring#3 stretch.
1712.52	1674	CC#1 stretch.
1751.36	1701	C=O #1 stretch.
1773.42	1716	C=O #2 & #3 asym. stretch.
1829.37	1782	C=O #2 & #3 sym. stretch.
2224.63	2129	alkyne stretch.

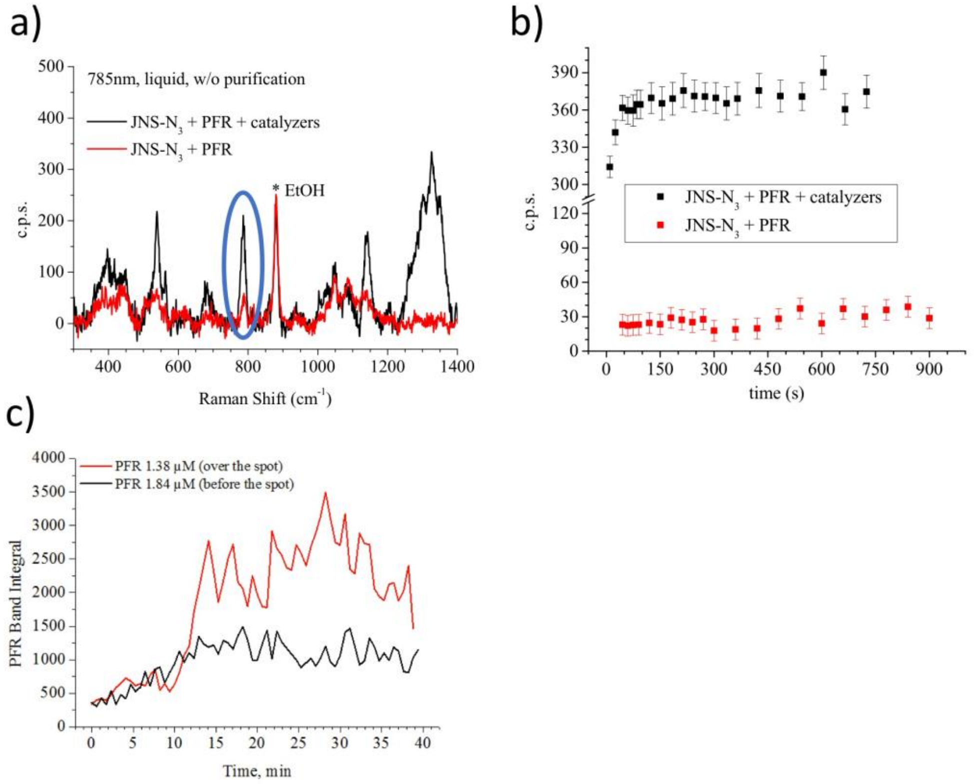

Figure S 8: a) JMNS and PFR are mixed in solution in the presence, or absence, of the catalysts (Cu(I) and ascorbate) needed to activate the cycloaddition reaction. Even without any purification step, the PFR signals can be obtained only in the first case. b) The same solutions of panel "a" were monitored in time, a plateau can be obtained in few minutes. c) JMNS and PFR were mixed, with the catalyst, in the microfluidic device and the SERS signal was monitored in time in correspondence of the magnet (i.e. where the spot forms) or just a few millimeters before. Thanks to the magnetic accumulation about 3-fold signal gain can be obtained.

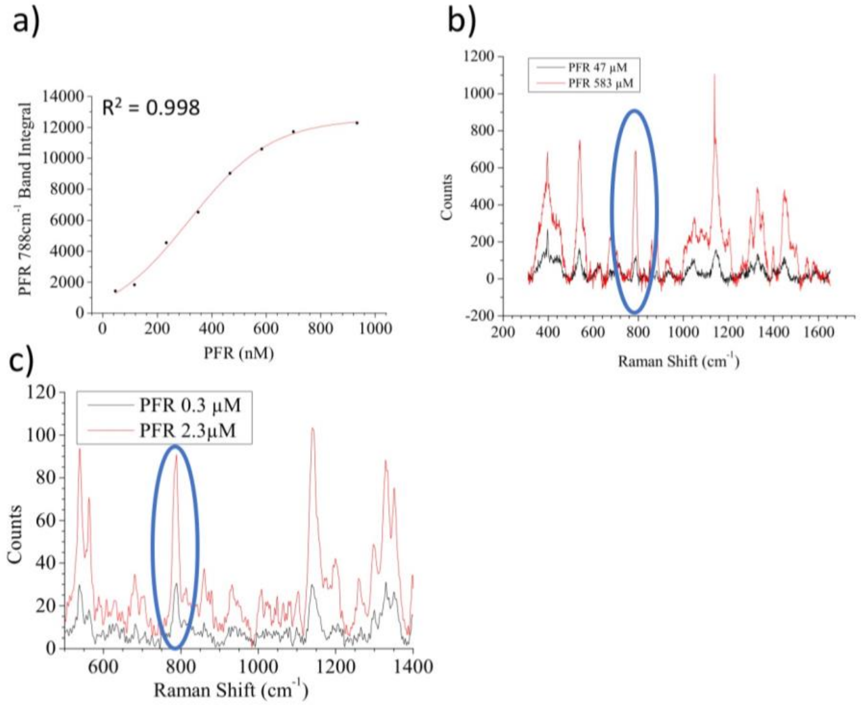

Figure S 9: a) Increasing the amount of PFR results in increasing SERS signals, the tests were performed in solution using always the same amount of JMNS; b) two spectra of panel (a) at low and high concentration; c) two spectra referring to the tests obtained with PFR and JMNS in the microfluidic device.

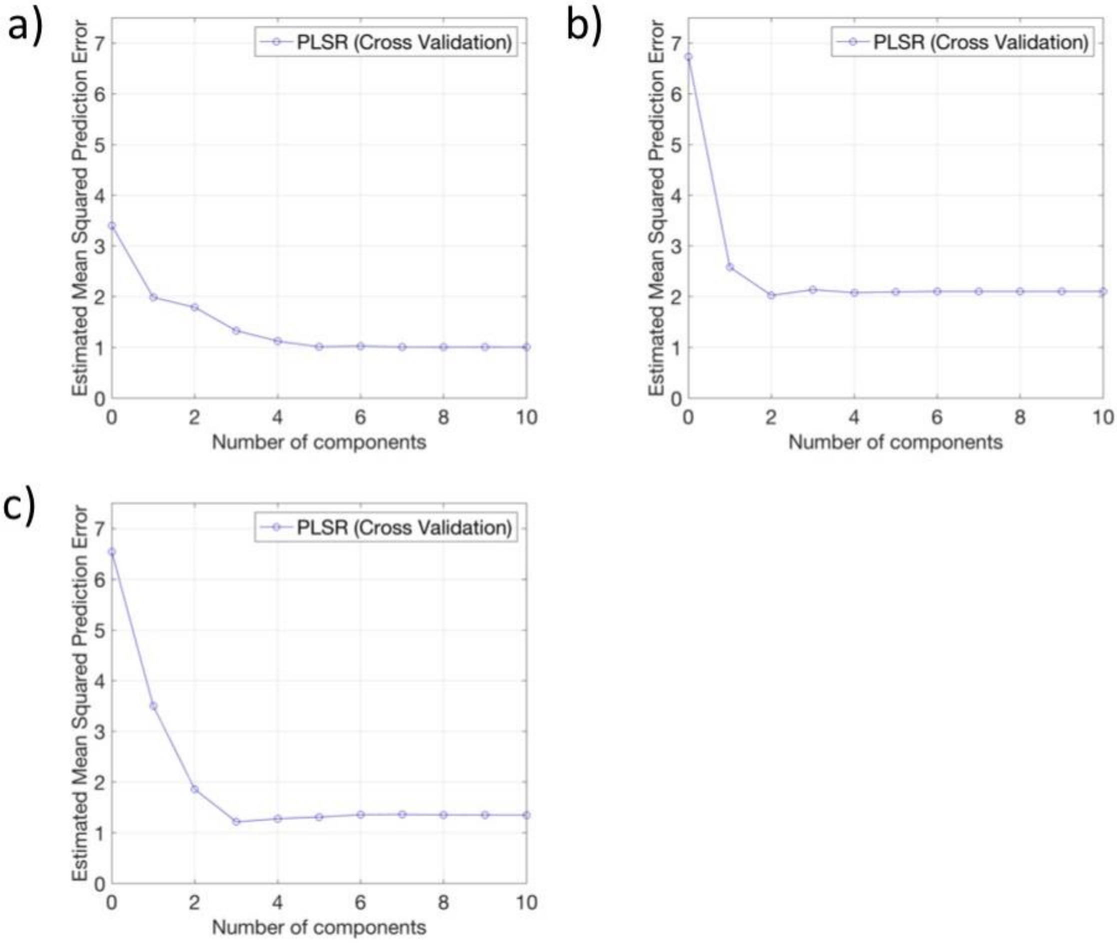

Figure S 10: Estimated Mean Squared Prediction Errors (MSE) as a function of the number of components used to build the prediction model in Partial Least-Squares Regressions. MSE values are calculated directly by the "plsregress" Matlab function applying a 10-fold cross-validation. a) MSE for Fluomioxazin competitive assay in the microfluidic device, b) MSE for water-spiked Erlotinib competitive assay in the microfluidic device, c) MSE for human plasma-spiked Erlotinib competitive assay in the microfluidic device.

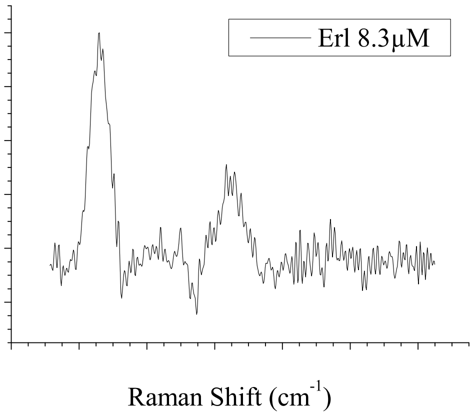

Figure S 11: SERS spectrum obtained by 8 µM of Erlotinig in human plasma. It can be evidenced how the PRF signals become progressively less recognizable at high Erlotinib concentration, a natural consequence of the competitive assay, where the highest PFR signals are obtained at the lowest analyte concentration. The band at about 860 cm^{-1}, ascribed to EtOH, is also less visible due to dilution.
